# The fate of nitrogen from different sources in a rice-wheat rotation system – A ^15^N labeling study

**DOI:** 10.3389/fpls.2023.1271325

**Published:** 2023-10-19

**Authors:** Wenxin Jia, Quan Ma, Li Li, Cunhu Dai, Min Zhu, Chunyan Li, Jinfeng Ding, Wenshan Guo, Xinkai Zhu

**Affiliations:** ^1^ Jiangsu Key Laboratory of Crop Genetics and Physiology, Agricultural College of Yangzhou University, Yangzhou, Jiangsu, China; ^2^ Co-Innovation Center for Modern Production Technology of Grain Crops, Yangzhou University, Yangzhou, Jiangsu, China; ^3^ Joint International Research Laboratory of Agriculture and Agri-Product Safety, the Ministry of Education of China, Yangzhou University, Yangzhou, Jiangsu, China

**Keywords:** N sources, rice-wheat rotation system, plant utilization efficiency, soil N residual rate, N loss rate, contribution ratio

## Abstract

High loss and low nitrogen (N) efficiency in agricultural production is severe. Also, ammonia volatilization and N leaching aggravated environmental pollution. The eutrophication of surface water and the emissions of N_2_O increased, hence green fertilization management urgently needs to be rationalized. Coordinating N supply from different sources has been shown to reduce environmental pollution. Therefore, this study was dedicated to clarifying the transport of N sources in the rice-wheat rotation system. The stable isotope tracer technology was used to label fertilizer (F), soil (T), and straw (J) with ^15^N, respectively. The utilization of N by crops (the N ratio in organs), as well as the residual N in soil and loss status, were measured. According to the potential of response to N, all the wheat cultivars were divided into groups with high (HNV) and low efficiency (LNV). The N contribution ratio showed that 43.28%~45.70% of total N accumulation was from T, while 30.11%~41.73% and 13.82%~24.19% came from F and J. The trend in soil N residue (T > F > J) was consistent with the above, while it was the opposite in N loss (T< F< J). The seasonal effectiveness showed that T achieved the highest N utilization efficiency (31.83%~44.69%), followed by F (21.05%~39.18%) and J (11.02%~16.91%). The post-season sustainability showed that T decreased the most in soil N residue (2.08%~12.53%), and F decreased the most in N accumulation (9.64%~18.13%). However, J showed an increase in N recovery rate (2.87%~5.89%). N translocation and distribution showed that N from different sources in grains was significantly higher than that in stems, glumes, and leaves. The ratio of HNV (75.14%~79.62%) was higher than that of LNV (71.90%~74.59%) in grain, while it was the opposite in other organs. Plant N accumulation, soil N supply, and straw N transformation were determined jointly by the three N sources, thus reducing N loss and N_2_O production. Therefore, the results will highlight the insights for constructing local N and emission reduction models.

## 1 Introduction

Wheat (*Triticum aestivum* L.) is the second largest food crop in China, occupying an important position in modern agricultural production due to its numerous elite varieties (Cui et al., 2023). With the increase in population density and the lack of natural resources, the demand for food has improved steadily. The linear increase in wheat yield has gradually become a crucial guarantee to solve the problem of food security (FAO, 2020; Tan et al., 2021). In agricultural production, N plays a dominant role in the growth and development of wheat (González-Cencerrado et al., 2020). In the rice-wheat rotation cultivation system, proper nitrogen application could improve the photosynthetic physiological potential of wheat by enhancing the synthesis of chlorophyll in leaves, which was conducive to photosynthesis and nutrient accumulation of crops (Falcinelli et al., 2022; Sharma et al, 2022). In addition, nitrogen has the potential to promote tillering and earing of wheat (Benincasa et al., 2017; Zörb et al., 2018). The first nitrogen absorption peak of wheat appeared in the tillering stage before winter, contributing to the formation of tillering and the improvement of the vitality index. The second peak was from the jointing to the booting stage when sufficient nitrogen could promote the differentiation of young spikes and the development of reproductive organs.

At present, the paddy rice-wheat double cropping area in China is mainly distributed in the middle and lower reaches of the Yangtze River, which is characterized by a humid climate and a shallow water table. The excessive application of nitrogen fertilizer was frequent, aggravating problems such as the excessive nitrate nitrogen content in groundwater and the increase of greenhouse gas (GHG) emissions (Sharma and Bali, 2018; Bhattacharya, 2019; Bhattarai et al., 2021). On the one hand, excessive application of nitrogen fertilizer could lead to the decline of ecological environment quality and economic yield of crops (Liu et al., 2018; Guo et al., 2021; Wan, 2021). On the other hand, nitrogen deficiency reduced the effective tillers at the seedling stage and spikes at maturity. The grain number per spike decreased in a later period due to the increase in floret degradation. Then, the nitrogen transferred to grains was reduced, which affected grain filling and 1000-grain weight, and ultimately led to a decrease in grain yield (Rivera-Amado et al., 2019; Pareja-Sánchez et al., 2020; Mahmood et al., 2022). Hence, rationally applying nitrogen fertilizer and improving its utilization efficiency is the prerequisite for the green and sustainable development of wheat (Cui et al., 2023).

After anthesis, nitrogen was mainly transferred from vegetative organs to reproductive organs, contributing to grain filling and nitrogen accumulation. Therefore, studies have been carried out on the rule of nitrogen translocation after anthesis under the high-yield system of crops. The tracer technique of ^15^N isotope labeling has been widely and accurately used in plant-soil systems, especially in the case of straw mulching (Xu et al., 2021). It is well known that there is a significant interaction between soil and residual straw in the rice-wheat rotation system (Rhymes et al., 2016; Wang et al., 2019). After the straw is returned to the field, it then returns nutrients to the soil through decomposition (Tan and Liu, 2015; Yang, 2023). Compared with traditional methods, nitrogen budget and straw nutrient decomposition rate were more accurately mastered under the combination of the two technologies. The dual goals of increasing crop internal nitrogen supply and reducing fertilizer input were achieved (Huddell et al., 2023). Soil nitrogen availability was improved and a stable high yield was obtained (Kaur et al., 2021).

At present, there are numerous studies on the source and fate of nitrogen, but the transfer principle of different nitrogen sources in the rice-wheat cultivation system for many consecutive seasons remains unclear. Therefore, a two-factor test based on the isotope tracing technology and straw returning technology was used to study nitrogen uptake, transport, and distribution in crops post-anthesis. According to the control variable method, the nitrogen utilization rate and seasonal change rate of crops were investigated. Both the external (temperature, humidity, etc.) and internal environments (pH, soil organic matter content, etc.) of crops have been considered in the experiment. Actually, we attempted to design a reasonable nitrogen management strategy for rice-wheat rotation fields, which may contribute to the sustainable development of agriculture and the gradual recovery of the ecological environment in the future.

## 2 Materials and methods

### 2.1 Description of the experiment site

The experiment was conducted at the Experimental Pot Farm of Jiangsu Provincial Key Laboratory of Crop Genetics and Physiology of Yangzhou University (119°25′E, 32°39′N) from 2021 to 2022. This area experiences a transition from a subtropical monsoon humid climate to a temperate monsoon climate. The average high temperature of the crop cultivation year was 22°CC, the average low temperature was 12°CC and the total precipitation was 638.1 mm. The typical soil in the test was sandy loam, which has been used for local conventional planting patterns in recent years. The soil’s basic fertility is described in **Table 1**. The tested rice variety Nanjing9108 was the main cultivar in Jiangsu Province, and the tested wheat varieties (Yangmai25, Yangfumai4, Shengxuan6, and Yangmai22) were bred by the Institute of Agricultural Sciences of Lixiahe District, Jiangsu Province.

**Table 1:** The soil properties before sowing at the rice experimental site.

Rice variety	pH	Organic matter(g kg^−1^)	alkali-Hydrolysable N(mg kg^−1^)	Available P(mg kg^−1^)	Available K (mg kg^−1^)
Nanjing9108	7.26	22.3	118.65	63.72	123.75

### 2.2 Experimental design

#### 2.2.1 Design of pot experiment in the first rice season

Sandy loam soil was used with approximately 55 kg per pot, totaling four pots. Nanjing9108 was transplanted in late June 2021 (**Table 1**). The planting specification was three rows per pot, five holes per row, and three plants per hole. The ratio of basal fertilizer : tillering fertilizer : spikelet-promoting fertilizer : flowering-preserving fertilizer was 5:1:2:2. Before transplanting, urea was applied at the N rate of 3 g pot^−1^. Phosphate fertilizer (P_2_O_5_) and potassium fertilizer (K_2_O) were applied as one-off basal fertilizer at 25 g pot^−1^ and 7.5 g pot^−1^, respectively. Urea was applied to approximately 1.7 g pot^−1^ at the 4~5 leaf stage; urea of 2.65 g pot^−1^ was applied at the penultimate one leaf stage. The ^15^N isotope was applied twice, with 56% as basal fertilizer and 44% as topdressing, a total of 36 g, and the isotope abundance was 60%. ^15^N topdressing was applied at the penultimate 2.5 leaf stage. A manual shelter was set up in the cultivation process. The other cultivation measures were referred to as the management measures of local experimental farmland. Immediately after maturity of 9108, the straw was taken and dried by natural light in the greenhouse. This step was to simulate the state of the straw from the harvest of rice to the sowing of wheat in the field trial. Also, the sample (only stems and leaves) was cut into approximately 10 cm for sealing. It was necessary to collect the soil in the growth stage of rice, sieve, and dry, and they were weighed at the same weight for standby. As two material factors before the second season of wheat planting, sieving was to ensure that the soil did not have excessive plate stubble, which had an unnecessary impact on the growth and development of wheat in the later stage.

**Figure 1:**
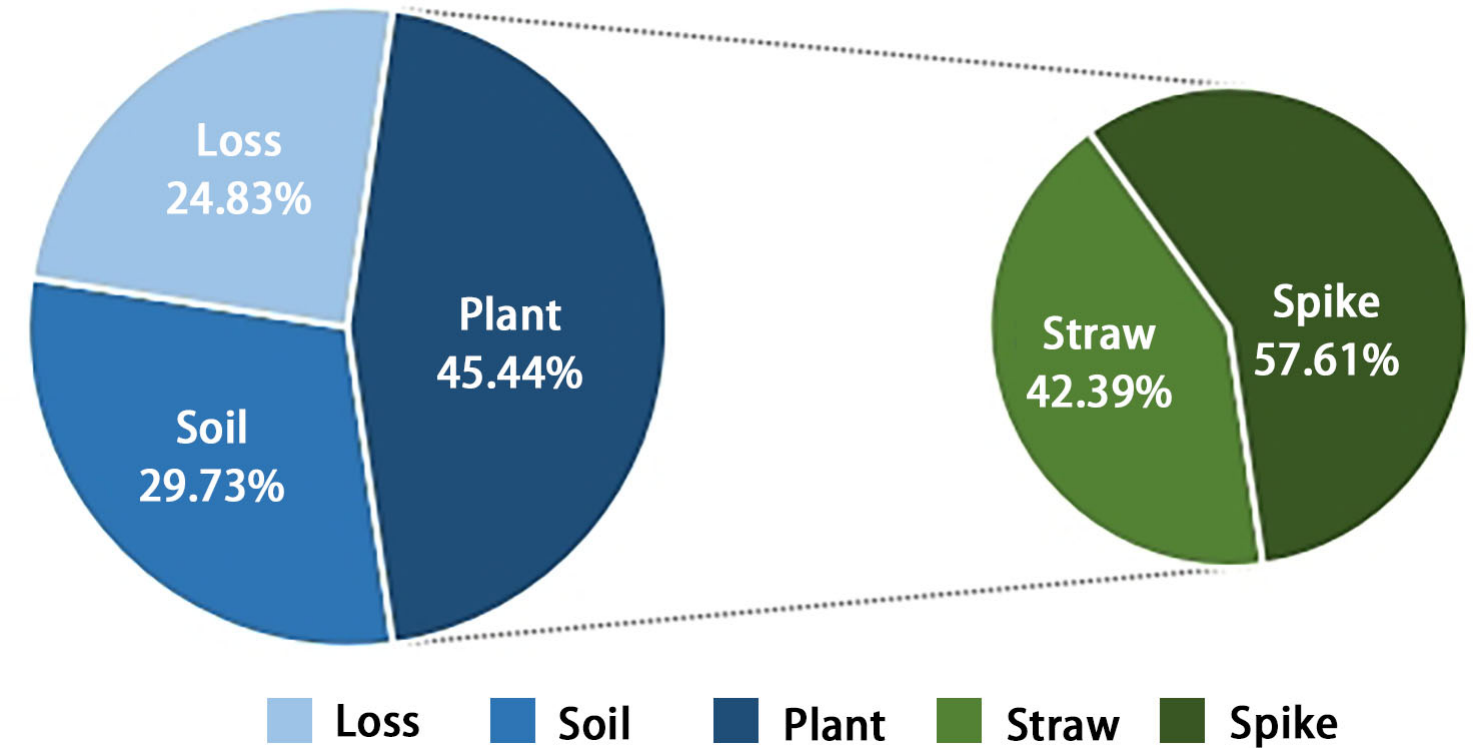
The comprehensive performance of ^15^N fertilizer nitrogen at maturity of rice. The soil represents the residual nitrogen that can be utilized in the next season, while the plant represents the nitrogen absorbed and utilized in the current season. It was divided into spike and straw. The percentage on the left represents the proportion of different nitrogen directions, while the right side represents the ratio of each component to the total absorption of the plant.

Rice was planted in the first season. The transfer and distribution ratio of the labeled nitrogen in the rice-soil system was studied in order to provide details of labeled straw and labeled soil for wheat (**Figure 1**). The transport of ^15^N isotope in the whole growth of rice was roughly divided into three directions: nitrogen loss rate (NLO), soil nitrogen residual rate (NSR), and nitrogen plant utilization rate (NPU). The final result was NPU > NSR > NLO. Among them, the ratio of isotopes absorbed by shoots from the application of nitrogen fertilizer was 29.48%, and NPU was 45.44%, which meant nitrogen accumulation in the spike was higher than that in the straw. The spike accounted for 57.61% of the total plant utilization, and the straw accounted for 42.39%. The proportion of soil residual nitrogen from nitrogen fertilizer was 19.5%, and NSR was 29.73%. The loss part was approximately 51.02%, and NLO was 24.83%. According to the principle of simulated field straw returning directly, the spike did not participate in further tests. The remaining straw stubble and residual fertilizer were treated as J and T for the next season, respectively. In addition, ^15^N in rice roots was calculated in the soil in order to improve test accuracy.

The conventional planting pattern of straw returning was simulated in the field. Three different nitrogen sources contained the same weight and type of straw, soil, and fertilizer, which ensured the accuracy of the control variables in the study. First, all the remaining soil from the first season was mixed evenly and set aside for subsequent experiments, and the dry humidity of each pot was equal to that of ^15^N-labeled soil. Secondly, the amount of straw required for each pot was converted according to the field ratio, and the same amount was weighed as a backup for ^15^N-labeled straw (fully mixed with the soil when used). Finally, in order to reduce the loss of isotopes in the experiment, a bottom-closed pot was used, which was also a prerequisite for achieving ^15^N-labeled fertilizer. The whole growth period of the experimental crop was ensured to be carried out under the rain shelter. This measure reduced the loss of consumables caused by extreme weather such as heavy rainfall and aimed to avoid large errors caused by surface runoff of ^15^N in the results.

#### 2.2.2 Design of pot experiment in wheat season

The previous crop was rice and the soil was sandy loam. A two-factor design was used. The main area was two high nitrogen-efficient wheat varieties: Yangmai25 and Yangfumai4, and two low nitrogen-efficient varieties: Shengxuan6 and Yangmai22 (**Table 2**). The sub-area was three different nitrogen sources (T, F, and J), with a total of 12 treatments (**Table 3**). Since there were some studies of related varieties in our research group, we cited their conclusions to continue the experiment (Ding et al., 2022). In early November 2021, artificial spot sowing was carried out in pots, with a sowing depth of 2 cm, 12 seeds per pot, 8 seedlings left, and 1 cm soil covered after sowing.

**Table 2:** The abbreviations of different nitrogen efficiency types, variety names, and treatments that will be repeatedly mentioned in the full text.

Species	Defined terms	Abbreviations
Wheat varieties	Shengxuan6	SX6
	Yangmai22	YM22
	Yangfumai4	YFM4
	Yangmai25	YM25
N variety classification	High nitrogen-efficient varieties	HNV
	Low nitrogen-efficient varieties	LNV
Treatments	^15^N labeled fertilizer	F
	^15^N labeled soil	T
	^15^N labeled straw	J
Nitrogen indicators	Plant Nitrogen Utilization Efficiency	NPU
	Soil Nitrogen Residual Rate	NSR
	Nitrogen Loss Rate	NLO
	Nitrogen Transport Efficiency (pre-anthesis)	NTE
	Nitrogen Contribution Rate to Grains (pre-anthesis)	NCR
	Nitrogen Harvest Index	NHI
Rice variety	Nanjing9108	NJ9108

**Table 3:** The soil properties before sowing at the wheat experimental site.

Treatment	pH	Organic matter(g kg^−1^)	alkali-Hydrolysable N (mg kg^−1^)	Available P (mg kg^−1^)	Available K (mg kg^−1^)
^15^N-labeled soil	8.07	14.75	84.8	63.02	82.68
^15^N-labeled straw and fertilizer	7.66	15.19	83.72	74.88	89.96

Phosphorus (P_2_O_5_) and potassium (K_2_O) fertilizers were a one-time basal application for 6.15 g pot^−1^ and 1.33 g pot^−1^, respectively. The nitrogen fertilizer application was basal fertilizer : tillering fertilizer : jointing fertilizer : booting fertilizer = 5:1:2:2, which was applied to 1.74 g pot^−1^, 0.35 g pot^−1^, 0.7 g pot^−1^, and 0.7 g pot^−1^, respectively. Basal fertilizer was mixed with water into the soil before sowing; tillering fertilizer was applied at the 4~5 leaf stage; jointing fertilizer was applied at the penultimate 2.5 leaf age, and booting fertilizer was applied at the penultimate 1 leaf age. All nitrogen fertilizers in F were replaced by ^15^N, and the same amount of urea was applied to T and J. In the cultivation process, a manual rain shelter was still set up. Except that the fertilizer was directly poured into the soil, other measures were based on the management measures of the local experimental field.

#### 2.2.3 Design of pot experiment in the second rice season

The previous crop was wheat and the soil was sandy loam. A single-factor randomized-block experimental design was adopted, and three treatments (T, F, and J) were set as before. Nanjing 9108 was still adopted, and the closed barrel remained unchanged. Phosphorous potash complex fertilizer (KH_2_PO_4_) was applied as basal fertilizer at one time for 1 g pot^−1^. The ratio of basal fertilizer : tillering fertilizer : spikelet-promoting fertilizer : flowering-preserving fertilizer was 5:1:2:2, which was applied to 1g pot^−1^, 0.2 g pot^−1^, 0.4 g pot^−1^, and 0.4 g pot^−1^, respectively. In addition, basal fertilizer was applied before transplanting; tillering fertilizer was applied at 4~5 leaf stage; spikelet-promoting fertilizer was applied at the penultimate 2.5 leaf age, and flowering-preserving fertilizer was applied at the penultimate 1 leaf age. The plants were transplanted in June 2022, and equidistant point insertion was used, with four points per pot and four plants per hole. Water replenishment and prevention of diseases were necessary according to the seedling situation later. Other management measures were the same as high-yield cultivation in the field.

#### 2.2.4 Systematic sampling and ^15^N determination

Ten shoots of rice with similar growth were taken in the frame at maturity. After 1 hour of deactivation at 105°CC, they were dried to constant weight at 80°CC. The shoots were divided into straw and spike for powder samples and then passed through 100 mesh (0.15 mm) for later use. At anthesis and maturity of wheat, four shoots with uniform growth were taken from three treatments respectively. After the shoots were separated, the same method as rice was used for the standby experiment. Meanwhile, the depth in 0~20 cm of soil was removed vertically from rice (maturity) and wheat (anthesis and maturity), repeating the process three times. The samples were mixed evenly and passed through 100 mesh (0.15 mm) after being dried and ground; 3~4 mg of each sample was weighed to be determined and wrapped into a regular rectangle (side length less than 5 mm) with tin paper. Then, the ^15^N abundance was measured with Isoprime 100 stable isotope mass spectrometer by putting them into the machine.

### 2.3 Statistical analysis

The ratio of nitrogen in each fate was determined and analyzed. NPU, NSR, and NLO represented plant utilization efficiency (%), soil residual rate (%), and loss rate (%) of nitrogen, respectively. The letters in parentheses were used to distinguish three nitrogen sources (**Table 2**). The ^15^N_uptake_, ^15^N_soil residue_, and ^15^N_total_ represented the total accumulation of ^15^N (g pot^−1^) in shoots, the residual amount of ^15^N (g pot^−1^) in soil, and the total input of ^15^N (g pot^−1^), respectively.


N F PU % = N 15 F uptake / N 15 F total

N F SR % = N 15 F soil residue / N 15 F total

$$N\;\left(F\right)\;LO\;\left(\%\right)\;=\;100-\;N\;\left(F\right)\;PU\;\left(\%\right)\;-\;N\;\left(F\right)\;SR\;\left(\%\right)$$

N T PU % = N 15 T uptake / N 15 T total

N T SR % = N 15 T soil residue / N 15 T total

$$N\;\left(T\right)\;LO\;\left(\%\right)\;=\;100-\;N\;\left(T\right)\;PU\;\left(\%\right)\;-\;N\;\left(T\right)\;SR\;\left(\%\right)$$

N J PU % = N 15 J uptake / N 15 J total

N J SR % = N 15 J soil residue / N 15 J total

$$N\;\left(J\right)\;LO\;\left(\%\right)\;=\;100-\;N\;\left(J\right)\;PU\;\left(\%\right)\;-\;N\;\left(J\right)\;SR\;\left(\%\right)$$

Nitrogen-related physiological indicators were also mentioned here. NHI, NTE, and NCR were used to represent the nitrogen harvest index, nitrogen transfer rate before anthesis (%), and the contribution rate of pre-anthesis transferred nitrogen to grains (%), respectively. N_grains_, N_anthesis_, N_maturity_, and N_maturity, veg_ were selected to represent nitrogen accumulation of grains (g pot^−1^), nitrogen accumulation of shoots at anthesis (g pot^−1^), nitrogen accumulation of shoots at maturity (g pot^−1^), and nitrogen accumulation of vegetative organs (g pot^−1^), respectively


$$NHI\;=\;N_{grains\;}\;/\;N_{maturity}$$

$$NTE\;\left(\%\right)\;=\;\left(N_{anthesis}\;-\;N_{maturity,\;veg}\right)\;/\;N_{anthesis}$$

$$NCR\;\left(\%\right)\;=\;\left(N_{anthesis}\;-\;N_{maturity,\;veg}\right)\;/\;N_{grains}$$

Excel 2016 and Origin 2022 were used to process data and draw pictures. DPS7.05 (Zhejiang University, Hangzhou, China) was used for statistical analysis, and the LSD method was used for dominance analysis.

## 3 Results

### 3.1 The flow direction of ^15^N isotopes during wheat maturity

**Figure 2:**
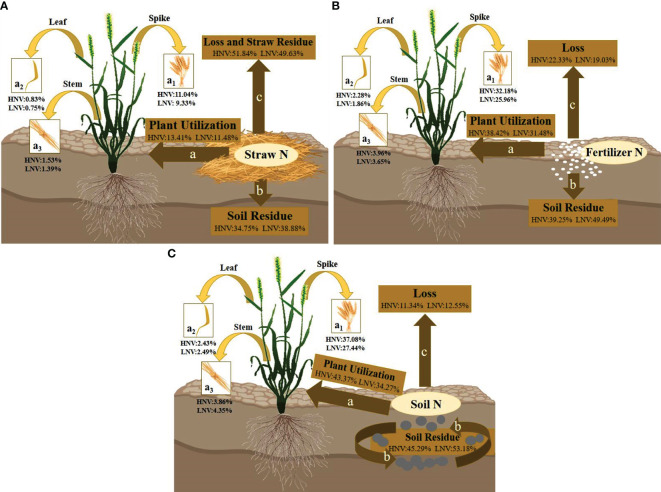
**(A–C)** Showed the overall nitrogen transport patterns of wheat at maturity when ^15^N-labeled with straw, fertilizer, and soil. a, b, and c represent three broad transport directions, (for ^15^N-labeled straw, the losses included residual nitrogen and losses of the straw itself); a_1_, a_2_, and a_3_ represent three narrowly defined transport flows to the plant, namely, to the spike, leaf, and stem. The percentage represents the relative numerical range of nitrogen loss rate, plant utilization rate, and soil residual rate in wheat with different nitrogen efficiency.

As can be seen from **Figure 2A**, when the nitrogen source was ^15^N-labeled straw, the nitrogen utilization efficiency of wheat at maturity was in the order of NLO > NSR > NPU (nitrification, denitrification, ammonia volatilization, and surface runoff were included in the losses, which was not mentioned below). It was speculated that because the straw was decomposed in the soil without adding a decomposition agent, the decomposition effect was terrible. Due to the slow release and decomposition of nutrients, most of the nitrogen in the straw still remained in itself, so the loss rate was higher in the current season. However, when the speed was accelerated in the later stage, a large amount of nitrogen could enter the soil, resulting in an increase in soil residual nitrogen.

**Figure 2B** shows that the nitrogen utilization efficiency of wheat at maturity was NSR > NPU > NLO under the treatment of ^15^N-labeled fertilizer. Since fertilizer nitrogen could be directly absorbed by crops or enter the soil as a supplement to the soil nitrogen pool without undergoing complex forms of transformation, its loss rate was relatively low.

According to **Figure 2C**, when the nitrogen source was ^15^N-labeled soil, the nitrogen fate of wheat with different nitrogen efficiency at maturity was NPU > NSR > NLO. The nitrogen supply capacity of the soil was affected by the C/N ratio, microbial activity, temperature, and humidity, but it did not prevent soil nitrogen from being the main source of nitrogen utilization for shoots.

By comparing three nitrogen sources, the plant utilization efficiency was found to be T > F > J. T was 1.14%~5.51% and 20.81%~30.65% higher than F and J, respectively. The soil residual rate was also T > F > J, with T being 2.72%~6.05% and 10.52%~15.42% higher than F and J, respectively. The loss rate was T< F< J, with T being 5.80%~11.53% and 36.23%~41.20% lower than F and J, respectively. The result of F may be due to the barrier of the fertilizer leaching downward so that most nitrogen was retained in the soil layer of 0~20 cm. Then, the utilization of fertilizer nitrogen was increased and the loss was declined. Meanwhile, the trend of the three treatments among the four varieties was basically the same. The utilization rate and loss rate were HNV > LNV, and the residual rate was HNV< LNV. A few data that did not meet this result appeared in T, which may be due to the fact that the nitrogen supply capacity of the soil was affected by the nitrogen pool and external environment compared to F and J.

The nitrogen distribution ratio of shoots at maturity was in the order of spike > stem > leaf (**Figure 2**). The trend of nitrogen absorption efficiency in different organs of wheat was identical in these three graphs. After anthesis, countless nitrogen flowed to the grains, while the leaves gradually turned yellow, or even partially withered. Ineffective tillers gradually died and decreased. Thus, the spike received the most nitrogen from the shoots.

### 3.2 Distribution of ^15^N in different organs of wheat

**Figure 3:**
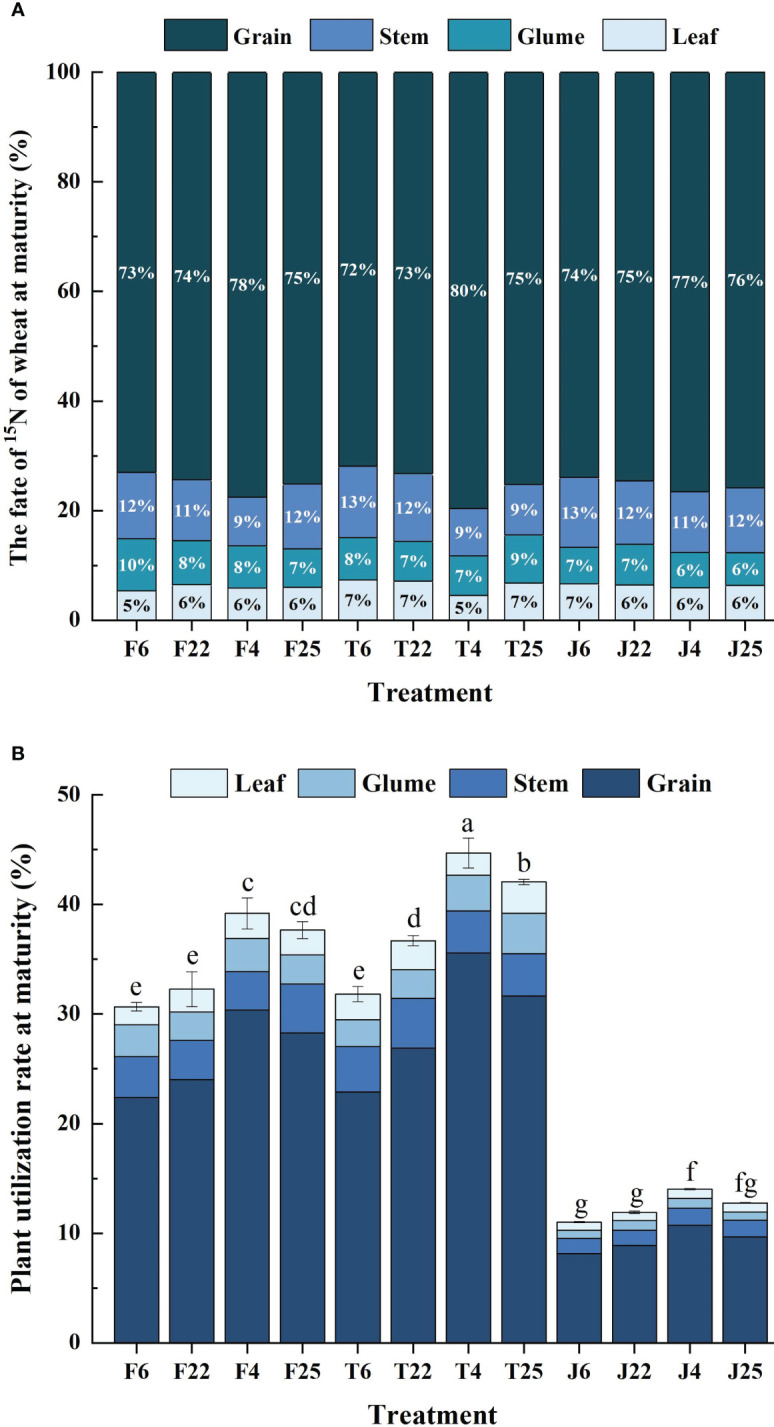
**(A)** Reveals the distribution ratio of nitrogen in the plant organs of wheat under all treatments in the study, including stem, leaf, grain, and glume. It represents the proportion of nitrogen in different organs in the shoots in order to compare the response potential of different organs to nitrogen. **(B)** Puts the proportion of organs into the nitrogen utilization efficiency of plants at maturity for overall comparison, which had advantages in observing the organ allocation of different nitrogen efficiency. Different lowercase letters indicate significant differences among the three cultivar groups at *P<* 0.05 **(B)**.

Isotope tracing technology was conducted to detect ^15^N in various organs of wheat at maturity (**Figure 3**). The ratio of ^15^N in grain, stem, glume, and leaf of wheat were 71.90%~79.92%, 8.63%~13.04%, 5.93%~9.53%, and 4.50%~7.39%, respectively (**Figure 3A**). There was no significant difference among the three nitrogen sources, indicating that nitrogen transport post-anthesis was regulated by the plant itself and was not affected by nitrogen sources.

The distribution ratio of nitrogen in ^15^N-labeled fertilizer, soil, and straw treatments was in the order of grain > stem > glume > leaf, and the proportion of grain was all higher than two-thirds of the total application of ^15^N (**Figure 3A**). The proportion of glume and leaf was basically lower than one-tenth of the total application of ^15^N (**Figure 3A**). Different nitrogen efficiency varieties had no difference in this rule, indicating that the nitrogen accumulated by shoots was mainly supplied to grain filling post-anthesis.

In addition, the nitrogen utilization efficiency of various organs in different varieties showed significant differences, basically being HNV > LNV and YFM4 > YM25 > YM22 > SX6 (**Figure 3B**). In terms of distribution ratio, different nitrogen efficiency varieties basically showed HNV< LNV among stems, leaves, and glumes, and HNV > LNV between grains (**Figure 3A**). It was indicated that different organs had different response abilities to nitrogen, and the nitrogen sensitivity of grains was higher. There was no significant difference under the same treatment in F and J for HNV, while the difference was significant in T, and the trend of LNV was consistent with HNV (**Figure 3B**). The effect of soil nitrogen on the utilization ability of different varieties was significantly higher than that of fertilizer and straw nitrogen.

### 3.3 Correlation analysis of different nitrogen indicators

**Figure 4:**
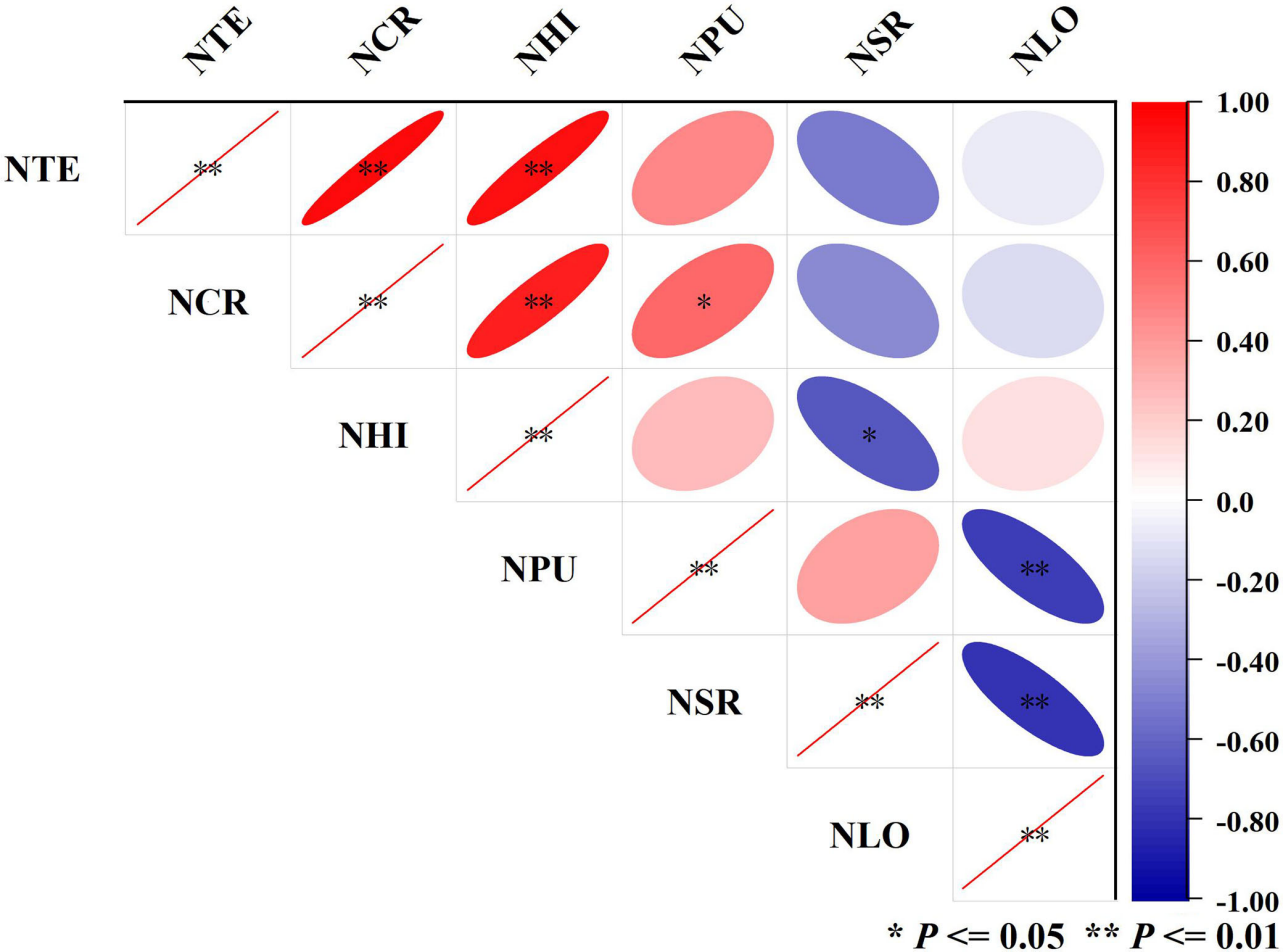
Summary of correlation of important nitrogen efficiency indicators. Blue at different depths represents a positive correlation, while red at different depths represents a negative correlation. The size of the ellipse reflects the size of the p-value. The larger the ellipse, the larger the absolute value of *P*, and the smaller the ellipse, the smaller the absolute value of *P*. * (*P*<= 0.05) represents a significant correlation, and ** (*P*<= 0.01) represents a very significant correlation.

Spearman correlation analysis was used to compare the correlation between different nitrogen physiological indicators (**Figure 4**), including NTE, NCR, NHI, NPU, NSR, and NLO (**Table 2**). There was a highly significant positive correlation among NTE, NCR, and NHI, indicating that nitrogen accumulation in the vegetative organs before anthesis was necessary due to the great significance for grain-filling, transport, and distribution of nitrogen after anthesis. More nitrogen accumulation before anthesis was beneficial for grains at the filling stage to fully absorb nutrients, and the three are interrelated and complementary. NPU showed no significant positive correlation with the above three factors, while NSR showed no significant negative correlation with them. Also, there was no clear pattern for the correlation analysis of NLO with them. There was no significant positive correlation between NSR and NPU, while NLO showed a highly significant negative correlation with NSR and NPU. The effect of plant nitrogen uptake and soil residue nitrogen were simultaneously responsive under the condition of equal nitrogen fertilizer application. It meant higher nitrogen accumulation determined higher accumulation and transport and they synergistically improved grain protein content at maturity.

### 3.4 The flow direction of ^15^N isotopes during rice maturity

**Figure 5:**
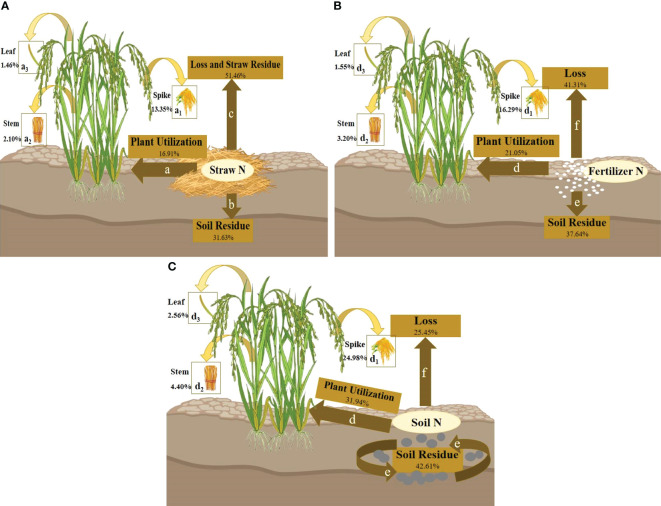
**(A–C)** Show the overall transport pattern of nitrogen from different sources (where nitrogen was the nitrogen remaining in the soil of wheat in the previous season, and the three sources were consistent with the above) for the third crop (rice) at maturity. Where d, e, and f represent three broad transport directions (for ^15^N-labeled straw, the loss included residual nitrogen and losses of the straw itself); d_1_, d_2_, and d_3_ represent three narrowly defined transport flows to the plant, namely, to the spike, leaf, and stem. The percentage represents the average value of nitrogen loss rate, utilization rate, and soil residual rate in wheat with different nitrogen efficiency.

The transport relationship of residual nitrogen between the third crop (rice) and the previous crop (wheat) is shown in **Figure 5**. The specific performance first appeared between different treatments. F and J showed NLO > NSR > NPU (**Figures 5A, B**), while T showed NSR > NPU > NLO (**Figure 5C**). Secondly, the different destinations of nitrogen were summarized as follows: plant utilization efficiency and soil residual rate were T > F > J, while loss rate was J > F > T (**Figure 5**). These patterns were consistent with the second season. Compared to F and J, T was 10.90% and 15.03% higher in plant utilization rate, 4.97% and 10.98% higher in soil residual rate, and 15.86% and 26.01% lower in loss rate, respectively (**Figure 5**). The distribution proportion of nitrogen among various organs of shoots showed spike > stem > leaf, which was also an inevitable result of multiyear crops (**Figure 5**).

Compared to the second season, the share of the three nitrogen destinations changed (**Figures 2**, **5**). For F and T, the utilization rate decreased by 13.90% and 6.88%, but the loss rate increased by 20.63% and 13.50%, respectively (**Figures 2**, **5B, C**). However, the plant utilization rate of J increased by 4.46%, and the loss rate remained basically unchanged, indicating that straw decomposition was more significant in the long term than in the short term (**Figures 2**, **5A**). All the nitrogen sources showed a small decrease in soil residual rate (5.19%~6.73%), which indicated that the nitrogen utilization rate could be greater than the recovery rate in the current season (**Figures 2**, **5**). During the two seasons, fertilizer nitrogen exhibited the greatest changes in nitrogen recovery rate while soil nitrogen showed the smallest. The results could be inferred that fertilizer nitrogen was unstable in the process of recycling and susceptible to the external environment, while straw nitrogen was suitable to provide reserve nitrogen for crops.

### 3.5 Different contributions of nitrogen sources among three nitrogen transport directions

**Figure 6:**
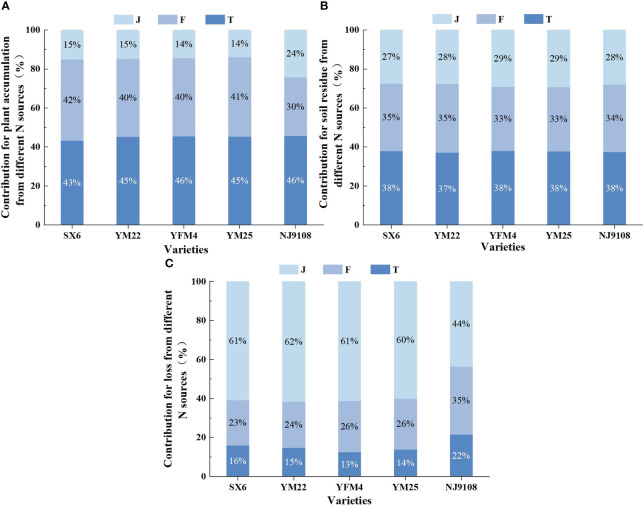
Effects of different nitrogen sources on plant nitrogen accumulation, soil nitrogen residue, and nitrogen loss in wheat and rice at maturity. **(A–C)** Represent the contribution rate of the three treatments to different transport directions.

The contribution rate for plant nitrogen accumulation and soil residual nitrogen from different sources were both T > F > J, while it was T< F< J of nitrogen losses (**Figure 6**). There were significant differences between different treatments (**Table 4**). The contribution rates for the nitrogen accumulation of shoots from soil nitrogen, fertilizer nitrogen, and straw nitrogen were 43.28%~45.70%, 30.11%~41.73%, and 13.82%~24.19%, respectively (**Figure 6A**). Also, soil residual nitrogen accounted for 37.18%~38.08%, 32.90%~35.20%, and 27.33%~29.21%, respectively (**Figure 6B**). Without special management, 12.56%~21.53% of lost nitrogen came from soil nitrogen, of which 23.21%~34.94% came from fertilizer nitrogen, and 43.53%~61.50% came from straw nitrogen (**Figure 6C**).

**Table 4:** Multivariate variance analysis of the contribution of the interaction between cultivars and nitrogen sources to the three nitrogen utilization indicators.

	NPU	NSR	NLO
V	**	**	**
S	**	**	**
V × S	**	**	**

** indicate a significant difference at the 1 % level (P<= 0.01). V and S refer to varieties and nitrogen sources, respectively.

There were different impacts on nitrogen flow among the three nitrogen sources in rice and wheat. The contribution rate of fertilizer nitrogen in plant utilization efficiency was 9.77%~11.62% lower than that in wheat, while that of soil nitrogen and straw nitrogen were 0.06%~2.42% and 9.20%~10.37% higher than that in wheat, respectively (**Figure 6A**). The ratio of straw nitrogen of rice in nitrogen loss decreased by 16.47%~17.97% compared with wheat, while soil nitrogen and fertilizer nitrogen increased by 5.47%~8.97% and 8.75%~11.73%, respectively (**Figure 6C**). There was no obvious trend in the distribution ratio of residual nitrogen in soil among different crops (**Figure 6B**).

### 3.6 Different contributions of nitrogen sources among three organs

**Figure 7:**
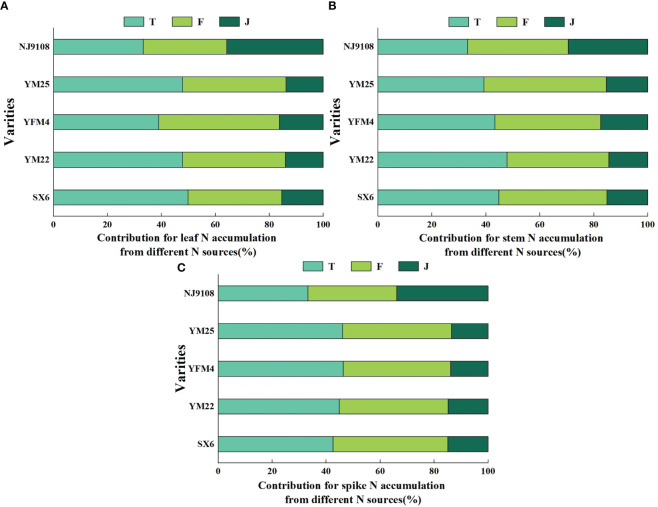
Effects of different nitrogen sources on leaf, stem, and spike in wheat and rice at maturity. **(A–C)** Represent the contribution rate of the three treatments to different organs.

As shown in **Figure 7**, there existed differences in the contribution of various organs among different nitrogen sources in the two crops. The organs of wheat showed a uniform trend of T > F > J. However, the rule for rice was quite opposite to the former. Nitrogen accumulation in stems of rice showed F > T > J, which showed J > T > F in leaves and spikes. The proportion of nitrogen accumulation in leaves, stems, and spikes of wheat from soil nitrogen was 39.01%~49.88%, 39.30%~47.82%, and 42.56%~46.28%, respectively. Similarly, the ratio of fertilizer nitrogen was 34.72%~44.74%, 37.72%~45.29%, and 39.83%~42.53%, respectively. There were differences in straw nitrogen, accounting for only 13.79%~16.24%, 14.46%~17.45%, and 13.60%~14.91%, respectively. Compared with that of wheat, the contribution rate of straw nitrogen for nitrogen accumulation in rice increased by 14.39%~1.99%, which was reduced by 0.49%~13.84% and 5.69%~16.56% for fertilizer nitrogen and soil nitrogen, respectively. Overall, the contribution rate of soil nitrogen in shoots was the highest, while the potential of straw nitrogen in the after-effect was significantly higher than that of soil and fertilizer nitrogen.

### 3.7 Comparison of trends in the second and third seasons

**Figure 8:**
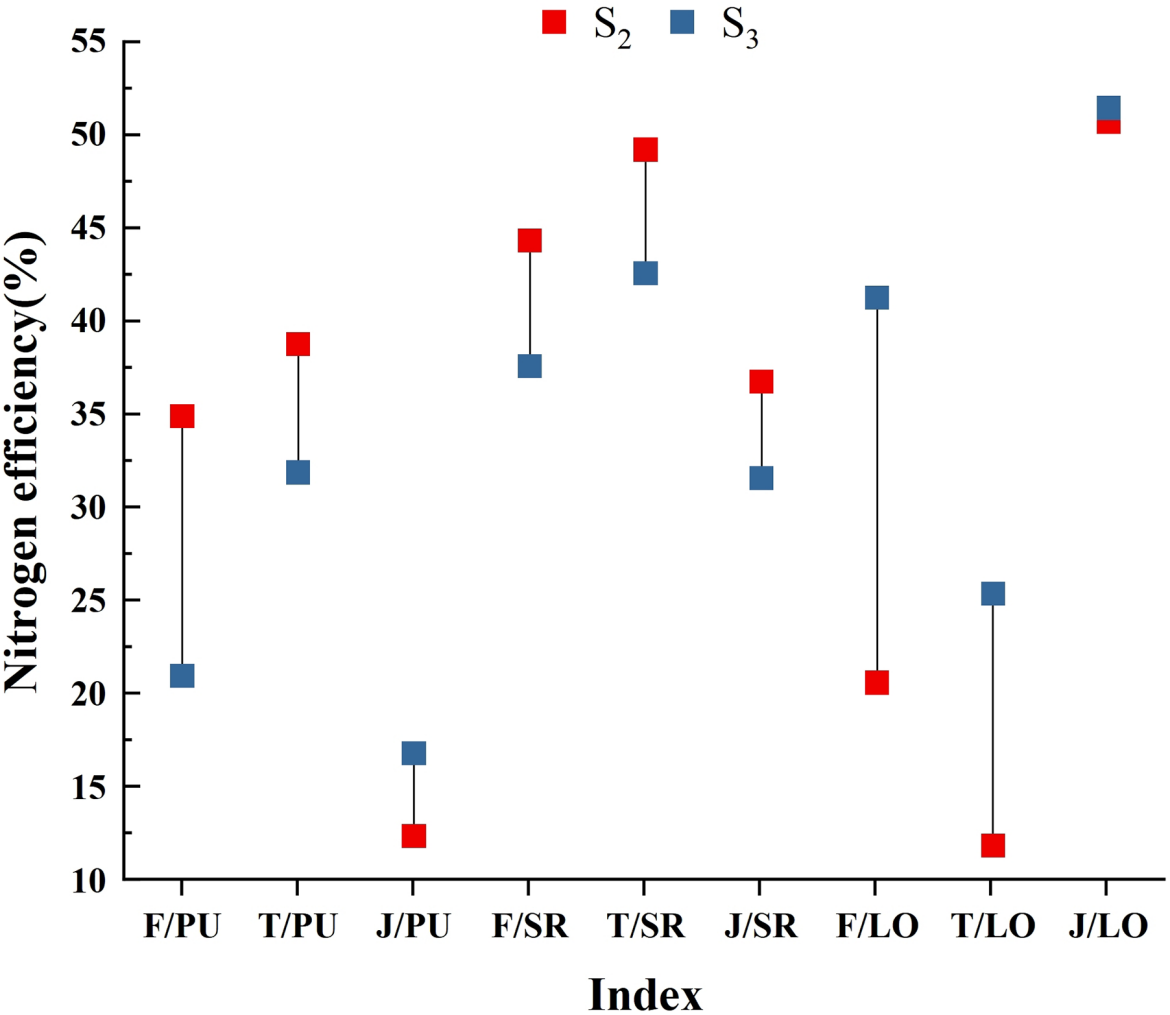
The figure shows the trend of nitrogen transport under different nitrogen sources. S_2_ and S_3_ represent the second and third quarters, respectively. The red square represents S_2_, the blue square represents S_3_, and the vertical line represents the difference in nitrogen efficiency between the two quarters under different treatments. Each indicator was named after ‘treatment/ratio’. The left side of the slash represents three treatments, namely, ^15^N-labeled fertilizer (F), ^15^N-labeled soil (T), and ^15^N-labeled straw (J). The right side of the slash represents nitrogen efficiency, namely, plant utilization efficiency (PU), soil residual rate (SR), and seasonal loss rate (LO).

In view of the nitrogen distribution patterns in the second and third quarters, a dumbbell chart was created (**Figure 8**). The results showed that the soil nitrogen of rice was in the order of S2 > S3, with 20.63%, 13.50%, and 0.73% less than that in wheat, respectively. In terms of loss rate, it showed that S_2_< S_3_, and the previous season of F, T, and J was 6.73%, 6.62%, and 5.19% lower than the subsequent season, respectively. However, there are differences in plant utilization efficiency among the three. F and T showed S_2_ > S_3_, with the previous season being 13.90% and 6.88% higher than the latter, while J showed S_2_< S_3_, with the previous season being 4.46% lower than the latter. The decomposition ability of straw was better in paddy fields than in wheat (paddy fields had sufficient water). In that way, nitrogen could be continuously released from the supplied straw in the later stage of utilization, which also depended on the mineralization ability of straw nitrogen. The decrease in loss rate was most significant under F and least significant under J. There was no significant difference in the decreasing trend of soil residual rate among them, only a slight decrease in J. The most significant decrease or increase in plant utilization rate was in F, while the smallest change was still in J. Differences between these trends were mainly caused by changes in crop characteristics, soil fertility, and even environmental conditions (temperature and humidity). The results also indicated that the seasonal performance of fertilizer nitrogen was the most unstable, of which soil nitrogen and straw nitrogen were more controllable in use. In addition, straw nitrogen had the greatest potential in the early operation of the rice-wheat rotation system.

## 4 Discussion

### 4.1 The contribution of different N sources to nitrogen transport in the crop-soil system

Crops maintain growth and development by absorbing nutrient elements from nitrogen fertilizers (Guo et al., 2022). The contribution ability of different nitrogen sources in the rice-wheat rotation multiple cropping systems was different, and there were commonalities and individuality among them. Soil nitrogen was composed of the primary nitrogen pool (soil native nitrogen) that was continuously exhausted without fertilizer nitrogen input and the self-fixed supplementary nitrogen pool (residual fertilizer nitrogen and straw nitrogen) after input of urea. It was generally divided into residual inorganic nitrogen and soil mineral nitrogen, which together determined the profit and loss of the soil nitrogen pool (Ye et al., 2022). Previous studies showed that 70.20%~79.27% of the nitrogen absorbed by crops at anthesis came from soil nitrogen, accounting for 45.0%~66.0% at maturity (Jia et al., 2011). It indicated that plants were most dependent on soil nitrogen when it was at a stable fertility level. In this paper, the statistics (43.28%~45.70% at maturity) were basically consistent with the previous results (**Figure 6**). The grain nitrogen accumulation also showed the rule, of which nitrogen translocation (73.1%~87.9%) before anthesis was much larger than nitrogen assimilation (12.1%~26.9%) after anthesis (Liu et al., 2020). Most of the pre-anthesis nitrogen translocation came from soil nitrogen, which also confirmed the physiological mechanism of soil nitrogen regulation crop response to nitrogen. In addition to the direct contributions, soil nitrogen also played an indirect role in nitrogen flow (Fang et al., 2022). The nitrogen absorbed from fertilizer nitrogen and straw residues was mineralized into organic nitrogen, thereby replenishing the endogenous supply of the soil nitrogen pool.

Fertilizer nitrogen had a legacy effect, changing the balance of the soil nitrogen pool directly or indirectly (crop residues) (Wytse et al., 2022). It showed a strong seasonal effect on nitrogen utilization, and the results in this study were basically consistent with other research. Fertilizer nitrogen that was not absorbed and stored by crops appeared as organic nitrogen in the soil, and then entered a depth of 0~100 cm in the soil with straw nitrogen, especially in the 0~40 cm soil layer in the form of mineralizable nitrogen (Chen et al., 2020). The high permeability of the 0~30 cm soil profile could lead to the high activity of straw and fertilizer nitrogen remaining in the surface layer (Hu et al., 2017). Also, the available amount could increase, which contributed greatly to the promotion of organic nitrogen mineralization in the soil and the supplement of the soil nitrogen pool. In this study, the soil residual rate of fertilizer nitrogen was approximately 37.64%~49.49% (0~20 cm soil layer) (**Figures 2B**, **5B**), which was higher than that in previous findings (24.5%~44.0% in 0~80 cm soil layer) (Jia et al., 2011; Shi et al., 2012). It was possible to calculate the crop type (wheat varieties with different nitrogen response efficiency), or the soil type (sandy loam, clay, etc.) could also be a major factor affecting the ability of nitrogen retention and remineralization of fertilizers, but the indirect effect of fertilizer nitrogen in the soil was still unconfirmed.

The effectiveness and contribution rate of straw throughout the entire growth period of plants seemed to be very low, while it has been proved that straw incorporation could increase crop yield (Bai et al., 2023). The sudden incorporation of crop straw into soil could lead to seasonal nitrogen deficiency in crops (Chen et al., 2016). It is possibly due to the modification of straw decomposition on soil physical properties, which encouraged the breakdown and reproduction of microorganisms, and then induced nitrogen fixation in the process (Huang et al., 2021; Sun et al., 2022). Therefore, straw nitrogen played an indispensable role in increasing the soil organic nitrogen pool, which was usually mineralized or immobilized when supplying crops (Zhang J. T. et al., 2017). The conclusion that the direction of ^15^N-labeled straw was soil residual rate > plant utilization efficiency also supported this viewpoint (**Figures 2A**, **5A**). Xu et al. (2021) found that the nitrogen utilization rate and loss rate of straw incorporation increased by 52.5% and decreased by 12.8%, respectively, compared with the control treatment. This situation also had an obvious influence on improving the harvest index and crop yields, which was consistent with our findings (**Figures 2A**, **5A**). Straw nitrogen has potential utilization value, reducing carbon footprint and increasing crop production (Dachraoui and Sombrero, 2020). Therefore, the efficient use of soil nitrogen was conducive to improving soil nitrogen supply capacity and promoting the effective implementation of nitrogen-saving goals, and it was of great significance in improving soil fertility.

In summary, fertilizer nitrogen and straw nitrogen maintained the balance of the soil nitrogen pool synchronously by directly converting it into organic nitrogen (Bai et al., 2023). Soil nitrogen and straw nitrogen simultaneously reduced gas emissions and promoted the implementation of nitrogen conservation and emission reduction (Liang et al., 2017; Li et al., 2021; Yang et al., 2022). Fertilizer nitrogen and soil nitrogen played a dominant role in plant nitrogen accumulation and transport at the same time. The three different sources of nitrogen synergistically reduced nitrogen loss, which was beneficial to improve nitrogen utilization efficiency under optimal nitrogen fertilizer management, and ultimately achieved the two goals of optimizing nitrogen fertilizer supply pathways and improving soil fertility.

### 4.2 Effects of different N sources on the availability of nitrogen in the current season and the sustainability in the following season

Some studies indicated that residual nitrogen in soil profiles (0~40 cm) had great potential (Chen et al., 2020). If not utilized by plants, most residual nitrogen would leach into soil layers of 1.2 m or even deeper in the form of nitrate (NO_3_^-^), greatly exacerbating losses (Richter and Roelcke, 2000). From the viewpoint of soil, nitrogen efficiency was regulated by soil depth and soil nitrogen residue to a great degree, as well as the strength of inherent soil productivity (Ren et al., 2023). Consistent with our findings, combined with a loss rate of ^15^N-labeled soil, the two seasons were 11.95% and 25.45%, respectively (**Figures 2C**, **5C**). The elaborate pot experiment design reduced the occurrence of losses, avoiding the effects of excessive irrigation or heavy rainfall (downward leaching or surface runoff) through closed barrels and concomitant rain shelters. This measure retained more effective nitrogen for subsequent crops and reduced the possibility of deep soil pollution (Zhang J. T. et al., 2017). When the fertilizer nitrogen was applied to the soil, although some of the nitrogen derived from fertilizer could enter the residual rice straw, it could return to the soil in a later stage (Wytse et al., 2022). This study confirmed the interpretation that the soil residual rate of wheat in the second season was in the order of T > F > J (**Figure 2**).

The initial application of fertilizer nitrogen was beneficial to ensure the nitrogen recycling efficiency of rice/wheat over several years while maintaining the stability of nitrogen transfer in the plant-soil system. In this experiment, ^15^N was applied in the first season of rice before transplanting, and then the residual fertilizer was retained and detected in the second season, confirming the effectiveness of nitrogen recovery in the later season (Yu and Shi, 2015). The plant utilization rate of F in the first season was 45.44% (**Figure 1**), while the rates in the second and third seasons were approximately 34.95% and 21.05% (**Figures 2B**, **5B**), which align well with the discovery by Smith and Chalk (2018), that the recovery rate decreased by 10% year by year in their study. According to their conclusions, the recovery rate of fertilizer nitrogen remaining in the soil in the first season was between 20% and 30%, which was slightly lower than our study. Water conditions and low soil nitrogen leaching rates were considered to be the reasons for this result (**Figure 2B**). In addition, there were some hypotheses that have been proved by previous studies that fertilizer nitrogen contributed more to nitrogen uptake by crops in the first few years after application (Wytse et al., 2022). Although the utilization rate showed a decreasing trend, there was no significant change in the soil residual rate (basically maintained at 40%); it seemed likely that there was still a potential for fertilizer nitrogen to be recovered in the later stage.

Other aspects that support the above views were that the ^15^N-labeled straw was designed in our experiment. It has been proved that the straw can be effectively used and recycled by crops for multiple seasons. The utilization rates of the two seasons were 12.45% and 16.91% (**Figures 2A**, **5A**), respectively, and the numerical gap under the ^15^N-labeled fertilizer was gradually narrowed (Xu et al., 2021). The loss rate of straw nitrogen in the last two seasons was 50.73% and 51.46% (**Figures 2A**, **5A**), respectively. There could exist two reasons for the higher results. The unrecovered straw nitrogen remained in the crop residue and was not decomposed, or was discharged in the form of gas (N_2_O or NH_3_) (Liu et al., 2021). In terms of nitrogen loss, it was possible that the undecomposed organic nitrogen was more than the emitted gaseous nitrogen (Yan et al., 2019). By comparing the lower loss rates of ^15^N-labeled soil and ^15^N-labeled fertilizer under the same nitrogen application management, as well as the increase of straw nitrogen in the utilization efficiency of two crops, this inference could be drawn (**Figures 2**, **5**).

Soil nitrogen and straw nitrogen could synergistically play a huge advantage in lowering production costs (mainly by cutting artificial nitrogen application) and reducing environmental pollution when it comes to economic and ecological benefits (Sun et al., 2022). However, we have not conducted thorough research on the standard measurement of environmental pollution indicators (the value of greenhouse gas emissions under different treatments and whether there are differences) (Huang et al., 2022; Zhang A. F. et al., 2017). Also, fertilizer nitrogen belonged to inorganic nitrogen, straw nitrogen belonged to organic nitrogen, and soil nitrogen belonged to organic and inorganic nitrogen. The high-efficiency utilization rate of soil nitrogen also relatively confirmed the significance of organic-inorganic fertilization in the nitrogen fertilizer model in the promotion of the agricultural industry nowadays (Choudhary et al., 2021).

## 5 Conclusion and prospect

Through the study of different sources of nitrogen, the proportion of different fates was considered; soil nitrogen had the largest contribution ratio to plant utilization and soil residue, followed by fertilizer nitrogen and straw nitrogen. According to the plant utilization rate, it could be judged that the after-effects of straw nitrogen and seasonal availability of soil nitrogen were both the strongest, while the stability of fertilizer nitrogen was the worst. Nitrogen accumulation and distribution ratio in shoots of rice and wheat both showed the most in grains and the least in leaves, regardless of the differences in nitrogen efficiency of crops. In summary, three nitrogen sources tested showed intensive characteristics and determined the necessity of nitrogen saving, quantified emission reduction, and multi-season recovery in crop production.

In the future, the practical application of this study will focus on the cultivation of soil fertility under the combination of straw returning and ^15^N stable isotope tracer technology. We hope that the need for fertilizer could be declined by making full utilization of straw nitrogen and soil nitrogen from the previous crops, as well as reducing production costs and reducing N_2_O emissions in the field. During this period, different straw returning methods (carbonized returning, total direct returning, adding decomposing agent returning, etc.) will also be used as a direction to extend the consideration of straw nitrogen. Through the optimization of management mode (reasonable C/N ratio and stable nitrogen recovery rate), high-yield and high-quality crop cultivation and sustainable development of ecological environment on the basis of nitrogen reduction will be achieved.

## Data availability statement

The original contributions presented in the study are included in the article/supplementary material. Further inquiries can be directed to the corresponding author.

## Author contributions

WJ: Data curation, Formal Analysis, Investigation, Methodology, Writing – original draft. QM: Investigation, Writing – review & editing. LL: Investigation, Writing – review & editing. CD: Investigation, Writing – review & editing. MZ: Project administration, Supervision, Writing – review & editing. CL: Conceptualization, Resources, Writing – review & editing. JD: Conceptualization, Resources, Writing – review & editing. WG: Conceptualization, Resources, Writing – review & editing. XZ: Project administration, Supervision, Writing – review & editing.
